# Changes in neuromuscular activation, heart rate and rate of perceived exertion over the course of a wheelchair propulsion fatigue protocol

**DOI:** 10.3389/fphys.2023.1220969

**Published:** 2023-10-18

**Authors:** Ursina Minder, Ursina Arnet, Erich Müller, Michael Boninger, Fransiska M. Bossuyt

**Affiliations:** ^1^ Neuro-musculoskeletal Functioning and Mobility Groupe, Swiss Paraplegic Research, Nottwil, Switzerland; ^2^ Department of Sport and Exercise Science, University of Salzburg, Salzburg, Austria; ^3^ Faculty of Health Sciences and Medicine, University of Lucerne, Lucerne, Switzerland; ^4^ Department of Physical Medicine and Rehabilitation, University of Pittsburgh, Pittsburgh, PA, United States; ^5^ Department of Health Sciences and Technology, Institute for Biomechanics, ETH Zurich, Switzerland

**Keywords:** spinal cord injury, shoulder pain, wheelchair propulsion, performance fatigability, perceived fatigability, neuromuscular activation

## Abstract

Shoulder pain is common in persons with spinal cord injury and has been associated with wheelchair use. Fatigue related compensation strategies have been identified as possibly impacting the development of shoulder injury and pain. The purpose of this study was to investigate the progression of performance fatigability (i.e., decline in objective measure of performance including neuromuscular activation and increase in heart rate) and perceived fatigability (i.e., increased perceived exertion) during a 15-min fatigue protocol including maximum voluntary overground wheelchair propulsion. Fifty participants with paraplegic spinal cord injury completed three 4-min rounds of wheelchair propulsion, separated by 90 s of rest, on a figure-8 course consisting of two turns and full stops per lap in their manual wheelchairs (ClinicalTrials.gov: NCT03153033). Electromyography (EMG) signal of five muscles acting on the shoulder joint, heart rate (HR), and rate of perceived exertion (RPE) were measured at the beginning and end of every 4 min of propulsion. Root Mean Square (RMS) and Mean Power Frequency were calculated from EMG data. There was a significant increase in %RMS of the pectoralis major pars sternalis and trapezius pars descendens, HR, and RPE with greatest changes during the first 4 min of the protocol. The observed changes in neuromuscular activation in only two of the shoulder muscles may impact muscular imbalances and the development of shoulder injuries and should be further studied. The current study gives clearer insight into the mechanisms of performance fatigability and perceived fatigability throughout a wheelchair propulsion fatigue protocol.

## 1 Introduction

Shoulder pain is a common problem in individuals with spinal cord injury (SCI) with a prevalence ranging from 36% to 70% (Gironda et al., 2004; Bossuyt et al., 2018) and chronic tendon degeneration is present in almost all wheelchair users (Arnet et al., 2022b; Jahanian et al., 2022). Socio-demographic factors like age (Alm et al., 2008) and gender (Bossuyt et al., 2018), characteristics of injury including level of injury (Sinnott et al., 2000), SCI severity and duration (Finley and Rodgers, 2004), and wheelchair use have all been associated with shoulder pathology and pain (Bossuyt et al., 2018). Even though previous studies showed that participation in wheelchair sports improves physical function and quality of life in individuals with SCI (McVeigh et al., 2009; Anneken et al., 2010) and Fullerton et al. (2003) describes possible protective mechanisms present in wheelchair athletes compared to their non-athletic counterparts the shoulder is a common site of complaints (Webborn and Emery, 2014; Heyward et al., 2017). Participation in wheelchair sports may lead to an increased risk of arm injury (Diaz et al., 2019). Karasuyama et al. (2022) states that factors and mechanisms of shoulder pain in wheelchair basketball are difficult to identify due to the presence of multiple potential factors. A systematic review by Heyward et al. (2017) came to the same conclusion and lists the following potential factors and underlying mechanisms for shoulder problems in wheelchair sports in general: overuse, weakness in shoulder adductors, internal and external rotators, decreased trunk control, poor driving posture in the wheelchair, poor scapular kinetics and muscular imbalances. Injury, pain and subsequent loss of mobility can influence a person’s independence and decreases quality of life (Gutierrez et al., 2007). Therefore, it is important to find the causes and develop preventive strategies.

Wheelchair propulsion is a demanding, repetitive activity which can result in fatigue (Mercer et al., 2006). It was indicated that repetitive mechanical loading of tendons alters the biochemical tissue responses possibly leading to tissue injury (Devkota and Weinhold, 2010). To this effect, acute (Porter et al., 2020) and long-term (Pozzi et al., 2022) supraspinatus tendon changes have been shown to occur in response to repetitive loading. These and other findings lead to the theory of repetitive mechanical load being one of the main factors to predict tendon related pain (Lewis, 2010). Repetitive fatiguing propulsion is a part of everyday wheelchair use as well as many wheelchair sports including wheelchair basketball, racing, rugby and tennis. With fatigue, the neuromuscular system is unstable and susceptible to injury (Pol et al., 2019). Arnoczky (2007) argue that fatigue or altered kinematics during repetitive load could lead to single or repetitive abnormal loading cycles causing damage to the tendon. With respect to the shoulder, fatigue can lead to different factors causing shoulder pain in people with SCI, such as alteration of the position of the humeral head (Chopp et al., 2010), alteration of scapulothoracic and glenohumeral kinematics (Ebaugh et al., 2006), decrease in shoulder proprioception (Lee et al., 2003) and acute changes in thickness of the supraspinatus and long biceps tendon (Bossuyt et al., 2020b).

Enoka and Duchateau (2016) describe fatigue as a global symptom rather than a decrease in performance of a specific structure. In their taxonomy two attributes of fatigue are acknowledged: performance fatigability and perceived fatigability. This definition of fatigue and fatiguability was adopted for the study at hand. Performance fatigability depends on the contractile capabilities of the muscles involved and the capacity of the nervous system to provide adequate activation and feedback for a given task. This can be quantified with changes in heart rate (HR) representing a global parameter of fatigability, muscular activation through electromyography (EMG: quantified by mean power frequency (MPF) and root mean square (RMS) (De Luca, 1997)), and isometric or dynamic force both local parameters for fatigability. Perceived fatigability on the other hand depends on the initial value and rate of change in subjective sensations and focuses on homeostasis and the psychological state which can be quantified with the rate of perceived exertion (RPE) (Borg, 1990) representing another global parameter. It remains unclear how these global and local parameters of fatigability relate in manual wheelchair users.

Investigating the effect of fatigue on shoulder muscles can provide further insights into injury risk. Although it would be ideal to observe the effect of fatigue during daily life or game play (van Drongelen et al., 2007), the measurements that can be performed in these settings are limited and the level of standardisation is lower compared to a laboratory setting. With fatigue being highly task dependent, it is important for potential test protocols to be close to activities of daily life or game play (Barry and Enoka, 2007). A figure-8 fatigue protocol (F8F) developed by Collinger et al. (2010a) is executed on level ground in the participants’ own wheelchair bringing the measurement of wheelchair propulsion induced fatigue closer to everyday activities. The protocol includes accelerations, deceleration, full stops and turns, which is closer to the conditions of everyday wheelchair propulsion and the training exercises included in several wheelchair sports and therefore potentially more relevant than fatigue measured during isometric or cyclic wheelchair propulsion movements (Collinger et al., 2010a). The short duration of F8F (15 min) and minimum requirement of equipment, make it a time-efficient and low-cost protocol that could be included in prospective cohort studies and assessments of wheelchair athletes to give, for example, guidance on readiness to return to play following an injury.

Studies using the F8F showed that the protocol resulted in (1) changes in biceps and supraspinatus tendon appearance (Collinger et al., 2010b; Bossuyt et al., 2020b), (2) shorter contact time in the first stroke of start-up propulsion (Bossuyt et al., 2020d), and increased neuromuscular activation in the M. pectoralis major, M. deltoideus and M. trapezius pars descendens as well as changes in stroke angle (Bossuyt et al., 2020a). To date, however, no study has investigated changes in muscle activity during the F8F itself limiting our understanding of the time-course of the development of performance and perceived fatigability. Therefore, the aim of this study was to investigate the progression in performance fatigability and perceived fatigability throughout the F8F and to investigate the relationship between local and global parameters of fatigability. We propose the following hypotheses: There will be significant changes in performance fatigability measured by mean power frequency (MPF) and root mean square (RMS) of the EMG signal of shoulder muscles and by HR throughout the F8F. Furthermore, there will be significant changes in perceived fatigability measured by RPE and significant correlation between local and global parameters of fatigability will be found.

## 2 Materials and methods

### 2.1 Study design and study population

This study, with a quasi-experimental one-group pretest-posttest design, was part of a larger project registered at ClinicalTrials.gov (Identifier: NCT03153033; Registration date: 15 May 2017) (Bossuyt et al., 2020a; Bossuyt et al., 2020c; Bossuyt et al., 2020d; Arnet et al., 2022a). Ethical approval was granted by the Ethikkommision Nordwest-und Zentralschweiz (Project-ID: 2017-00355). A total of 50 participants were recruited via the Swiss Spinal Cord Injury Cohort Study (SwiSCI) database. Included for participation were individuals with diagnosed paraplegia (injury level T2 or below), nonprogressive traumatic or atraumatic SCI, 1 year or more after completion of inpatient rehabilitation, aged between 18 and 65, daily use of a manual wheelchair and no required support for moving around more than 100 m in a wheelchair. Exclusion criteria were as follows: patients in palliative care, congenitally caused SCI, neurodegenerative disorders or Guillain-Barré syndrome, pain in the upper limbs that restricts wheelchair propulsion, past shoulder, elbow or wrist fractures or dislocations that cause symptoms, history of cardiopulmonary problems that could be worsened by demanding physical activity.

### 2.2 Procedure

Written informed consent was obtained from all patients prior to the 4 h testing session which took place in the biomechanical laboratory at the Swiss Paraplegic Research. Participants were instructed to avoid strenuous exercise 48 h before the testing day. After familiarization with the procedures, several assessments were conducted before, during and after the F8F as part of the overall project and not further analyzed for this paper. The standardized procedures prior to the F8F lasted for about 3 h and included preparations for EMG and kinematic measurements, completion of questionnaires, wheelchair propulsion (15 m sprint and propulsion on a treadmill) and ultrasound examination of the shoulder. For further details on these assessments, which will not be part of the analysis in the current study, please consider the following publications: Bossuyt et al. (2020a); Bossuyt et al. (2020b); Bossuyt et al. (2020d); Arnet et al. (2022a). These previous studies published results from this dataset including changes in treadmill propulsion biomechanics and ultrasound measures of shoulder tendons and acromiohumeral distance before and after the F8F.

### 2.3 Figure-8 fatigue protocol

**FIGURE 1:**
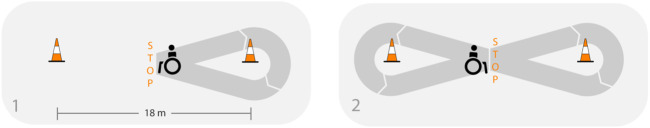
Adapted from Arnet et al. (2022a), licensed under CC BY 4.0: Illustration of one lap of the figure-8 protocol.

The F8F consists of three repetitions of 4 min of wheelchair propulsion along a figure-8 shaped course with 90 s of rest in between (Collinger et al., 2010a). Two cones were placed 18 m apart and the starting point as well as the direction of the 2 turns were marked on the floor. Participants were instructed to propel as many laps as possible in 4 min. Every lap includes a right and left turn and 2 full stops at the crossing point (Figure 1). Instructions and motivational input given during the protocol were standardized.

### 2.4 Data collection

Socio-demographic, personal and injury related information were self-reported. Muscle activity was recorded during the first and last 30 s of each 4 min propulsion of the F8F with the use of surface EMG (Telemyo 2400T Direct Transmission System, 305 Noraxon, Inc. United States) of the M. biceps brachii, M. pectoralis major pars sternalis, M. deltoideus pars acromialis, M. trapezius pars descendens and pars ascendens on the non-dominant side in accordance to the SENIAM guidelines (Hermens et al., 2000). The non-dominant side was chosen to minimize the influence of handedness. A wireless system and self-adhesive snap bipolar AG/AgCL surface electrodes were used to record EMG data at 1,500 Hz. HR was measured using a heart rate monitor Polar H800 (Polar, Electro, Finland) and RPE was captured with a 20 point Borg scale (Borg, 1990). Both parameters were measured and written down before and after every 4 min session of the F8F.

### 2.5 Data analysis

Raw EMG signals were offset corrected, rectified, filtered with a high pass (20 Hz) and low pass (3 Hz) 3^rd^ order Butterworth filter. Smoothing was executed using moving average (0.05 s 50% overlap) (Supplementary Figure S1). This generated linear envelope was used to calculate the RMS which is expected to increase with fatigue (De Luca, 1997). The MPF, which is expected to decrease with fatigue (De Luca, 1997), was calculated using the raw EMG data. RMS and MPF of all six measurements of the five muscles were calculated with a short-time Fourier transformation (MacIsaac et al., 2001). RMS and MPF signals were calculated as a percentage of the initial RMS and MPF.

### 2.6 Statistical analysis

Statistical analysis was done with Statistical Package for the Social Sciences (SPSS Statistics 26, IBM). The hypotheses were tested with one-way repeated measure analysis of variance (ANOVA). The dependent variables were MPF and RMS and the independent variable was time (6 time points representing the beginning and end of every 4 min of the F8F). If Mauchly’s test of sphericity was significant, Greenhouse Geisser Corrected *p*-values were used. If statistical significance was found, pairwise comparisons with Bonferroni corrections were used. Correlation coefficients (Spearman- or Pearson-Test) between changes in HR, RPE and RMS of muscles with significant effects for time, were calculated and interpreted according to the recommendation of Dancey and Reidy (2007) defining values 0.1 to 0.3 (−0.1 to −0.3) as weak, 0.4 to 0.6 (−0.4 to −0.6) as moderate and 0.7 to 0.9 (−0.7 to −0.9) as strong correlations. The level of significance was set to *p* < 0.05.

## 3 Results

Due to technical problems with the EMG, 7 of the 50 participants had to be excluded. Data of 43 participants were investigated (age: 50.2 ± 10.1 years, weight: 72.1 ± 13.3 kg, TSI: 27.1 ± 11.6 years, cause of injury: 93% traumatic, completeness: 84% incomplete, lesion level: 42% T2-T6, 42% T7-T12, 16% L1-L5, dominant hand: 93% right handed). Subject characteristics of the included participants did not significantly differ from the excluded participants except with regards to completeness of injury with 84% of the included participants had an incomplete injury compared to 43% in the excluded group (Table 1). During the F8F protocol, the participants completed 9.6 ± 1.4 laps (mean, SD) in the first bout of the protocol, 9.8 ± 1.4 in the second bout and 9.9 ± 1.5 in the last bout.

**TABLE 1:** Characteristics of the included participants and the participants excluded due to technical problems.

	Included (N = 43)	Excluded (N = 7)
Age (years)	50.2 ± 10.1	52.6 ± 6.8
Height (m)	173.9 ± 7.7	172.3 ± 9.2
Weight (Kg)	72.1 ± 13.3	74.3 ± 14.2
Time since injury (years)	27.1 ± 11.6	24.0 ± 12.5
Age at injury (years)	23.1 ± 10.1	28.6 ± 9.4
Sex (n, %) male	35 (81%)	4 (57%)
female	8 (19%)	3 (43%)
Cause of injury (n, %) traumatic	40 (93%)	6 (86%)
non-traumatic	3 (7%)	1 (14%)
Completeness (n, %) complete	7 (16%)	4 (57%) *
incomplete	36 (84%)	3 (43%) *
Dominant Hand (n, %) right	40 (93%)	7 (100%)
left	3 (7%)	0
Lesion level (n, %) T2-T6	18 (42%)	2 (29%)
T7-T12	18 (42%)	4 (57%)
L1-L5	7 (16%)	1 (14%)

* indicating significant difference between included and excluded group, *p* < 0.05.

**FIGURE 2:**
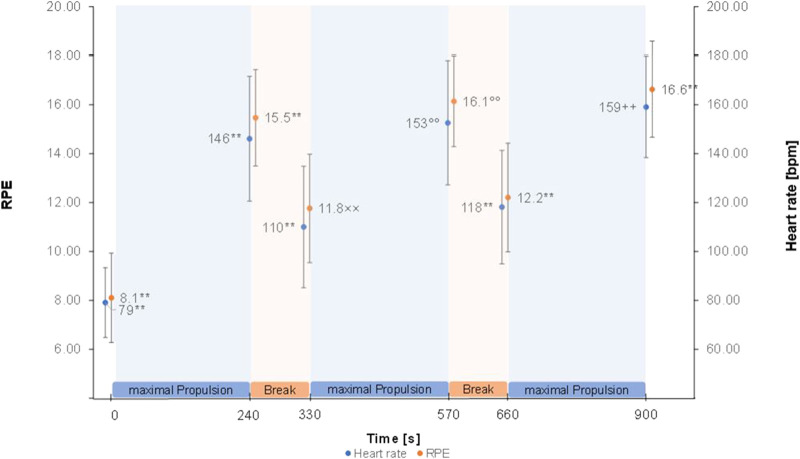
Heart rate and RPE means ± SD over the figure-8 fatigue (F8F) protocol. ** marking highly (*p* < 0.001) significant difference to all other points of measurement. ° marking highly significant difference to all other points of measurement except with 900 s ×× marking highly significant difference to all other points of measurement except with 660 s ++ marking highly significant difference to all other points of measurement except with 570 s.

**FIGURE 3:**
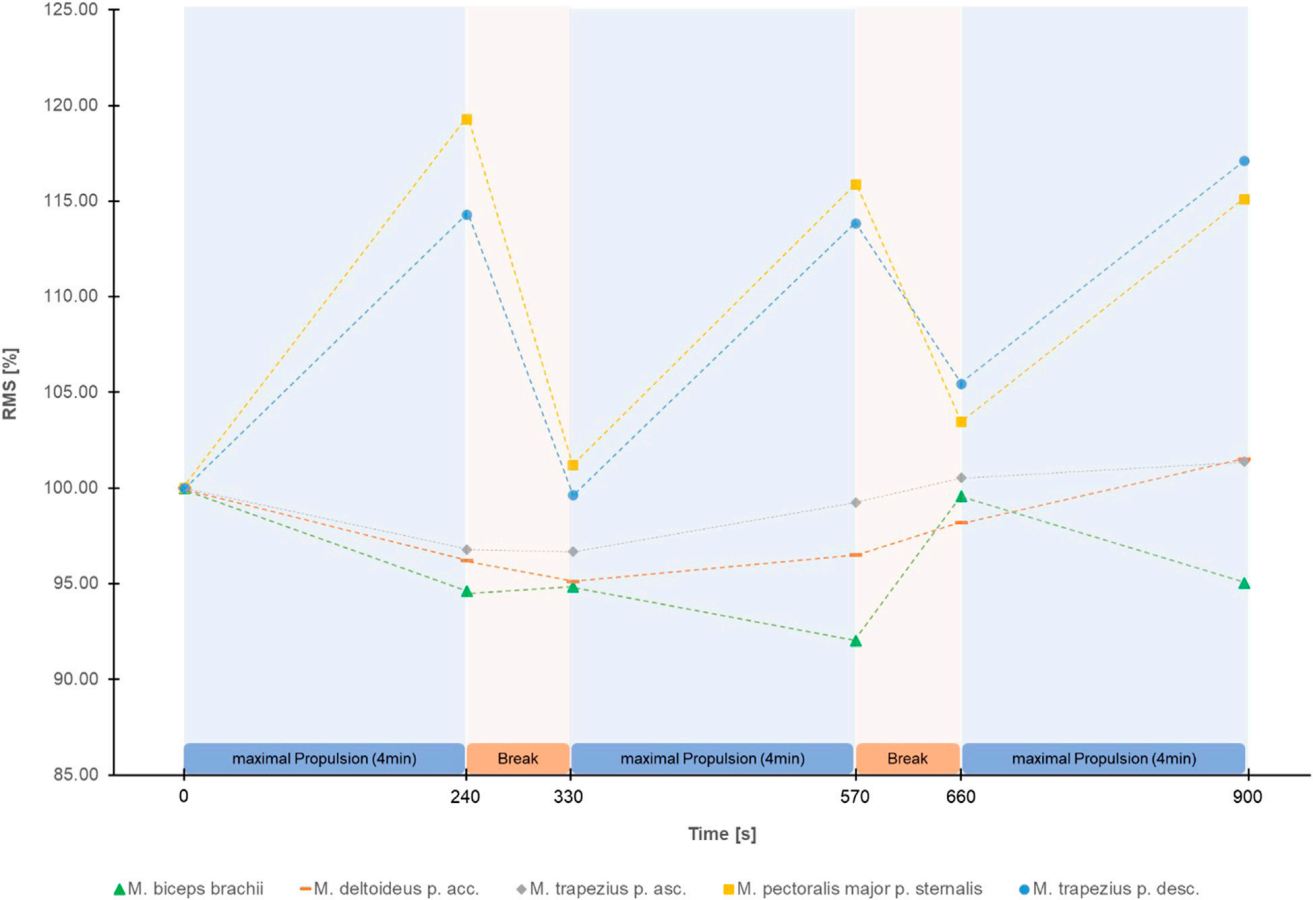
Mean Root Mean Square (RMS) in percentage of the start RMS of the EMG signal of the 5 shoulder muscles measured during the figure-8 fatigue (F8F) protocol.

The Greenhouse Geisser corrected repeated measure ANOVA’s showed a significant effect of time for HR (F (2.58, 105.58) = 378.88, *p* < 0.001, partial η^2^ = 0.90), RPE (F (2.81, 117.99) = 199.12, *p* < 0.001, partial η^2^ = 0.83) (Figure 2), RMS of M. trapezius pars descendens (F (1.83,71.31) = 9.01, *p* < 0.001, partial η^2^ = 0.19) (Supplementary Figure S2) and RMS of M. pectoralis major pars sternalis (F (1.90, 72.05) = 8.50, *p* < 0.01, partial η^2^ = .18) (Supplementary Figure S3). Post-hoc test results are reported in Figure 2 and Supplementary Figure S2, S3. No significant effects were found for the RMS of M. deltoideus pars accromialis (F (2.42,94.55) = 1.65, *p* = 0.191), M. trapezius pars ascendens (F (3.09, 120.57) = 0.946, *p* = 0.423), and M. Biceps brachii (F (3.67, 135.86) = 1.85, *p* = 0.129) (Figure 3). No significant effects were found for MPF of the five measured muscles.

Weak to moderate correlations were found between changes in HR and RPE as well as between changes in RMS of M. pectoralis pars sternalis and RMS of M. trapezius pars descendens. Table 2 gives an overview of the significant correlations. No other significant correlations (e.g., between changes in HR and changes in RMS) where found.

**TABLE 2:** Spearman Rho for changes throughout and total change throughout the F8F.

		Propulsion 1 (0s–240s)	Rest 1 (240s–330s)	Propulsion 2 (330s–570s)	Rest 2 (570s–660s)	Propulsion 3 (660s–900s)	Total change (0s–900s)
Correlations between HR and RPE	R	0.395*	0.333*	0.310*	0.158	0.333*	0.322*
	Sig.	0.008	0.022	0.036	0.288	0.022	0.035
Correlation between RMS of TD and RMS of PM	R	0.460**	0.434**	0.397*	0.368*	0.523**	0.538**
	Sig.	0.003	0.005	0.010	0.020	0.000	0.000

(* marking a weak correlation (0.1–0.3), ** marking a moderate correlation (0.4–0.6)). Abbreviations: HR, heart rate; RPE, rate of perceived exertion; RMS, root mean square; TD, M. trapezius pars descendens; PM, M. pectoralis major pars sternalis.

## 4 Discussion

This study investigated changes in local and global parameters of performance over the duration of the F8F. Significant changes indicating fatigability throughout the F8F were registered in HR, RPE and RMS of M. trapezius pars descendens and RMS of M. pectoralis major pars sternalis. More specifically the protocol showed changes in parameters of performance fatigability with an increase in mean heart rate from 79 ± 14 bpm right before the protocol to 159 ± 21 bpm right after the protocol and the expected HR and RMS patterns of M. trapezius pars descendens and M. pectoralis major pars sternalis during the protocol (increase at the end of every 4 min active propulsion phase, decrease after the 90 s rest). Furthermore, an increase in RPE as a parameter of perceived fatigability was observed from very light exertion to very hard exertion.

Interestingly, greatest changes in RMS were observed over the first 4 min of the F8F. The correlations between changes in RMS of the M. pectoralis major and RMS of the M. trapezius pars descendens showed that these two muscles fatigue simultaneously rather than sequentially. The correlation of changes in HR and RPE although weak further demonstrate the association of more global measures of performance and perceived fatigability. Nevertheless, these global measures of fatiguability did not correlate with the changes in RMS, a local measure of performance fatiguability.

An increase in RMS is related to an increase in estimated amplitude of force twitches of motor units and further demonstrates the size principle of recruitment of motor units present in fatiguing activities. In the unfatigued state, slow motor units are recruited, then during the course of activity or with increased intensity larger and faster motor units follow (De Luca, 1997). As the smaller motor units fatigue, fine coordination of the affected muscles could decrease, possibly leading to harmful changes. In case of wheelchair propulsion, scapulothoracic and glenohumeral movement patterns may be affected (Chopp et al., 2010). Fatigue in only a part of the shoulder stabilizing muscles could lead to/or increase muscle imbalances leading to more stress on the rotator cuff muscles and promoting shoulder injury and pain (Burnham et al., 1993). These findings underline the importance of exercise programs targeting strengthening and thus reducing the fatigability of the scapular stabilizers (Van Straaten et al., 2014). Furthermore, with muscle imbalance being one of the possible underlying factors for increased risk of shoulder problems in wheelchair sports (Heyward et al., 2017) further examination of fatiguing propulsion in sport specific settings is crucial.

No consistent patterns regarding fatigue on a muscular level were observed for the M. deltoideus pars acromialis, M. trapezius pars ascendens, and M. biceps brachii. Slowik et al. (2016) found high variations in the activity of the M. deltoideus pars acromialis between the different propulsion techniques especially during the recovery phase of each wheelchair push. The unclear results regarding fatigue of the M. deltoideus pars acromialis could be explained by this wide variety of movement techniques during the recovery phase. Subgroup analyses comparing different propulsion and recovery phase techniques could give further insight in the topic.

The lack of changes of MPF data during the F8F may be related to the discontinuous nature of the used protocol including and varying technical approaches in propulsion, breaking and turning between different participants. MPF is recommended for the analysis of isometric or strictly cyclic tasks (MacIsaac et al., 2001). Other investigations with the same participants showed a reduction in MPF for M. Pectoralis major, M. deltoideus, M. trapezius pars ascendens and M. biceps brachii during cyclic wheelchair propulsion on a treadmill after the F8F (Bossuyt et al., 2020a). Furthermore differences to the findings of Bossuyt et al. (2020a) regarding RMS and MPF values and level of changes can be explained by the temporal differences in the measurement protocols. In the study at hand, EMG data was collected during the protocol and the first measurement of the protocol was taken as 100% for RMS and MPF. Bossuyt et al. (2020a) analysed fatigue using data from testing pre and post protocol treadmill tests at fixed power output and investigated absolute values of the MPF. Measuring during the protocol might ignore that some participants where already fatigued due to the preparation procedure and previous measurements (e.g., participants performed a sprint test and wheelchair propulsion on the treadmill). Furthermore, it is questionable if changes in MPF indicating fatigue would take more time after exposure to manifest.

Several limitations need to be acknowledged. First, although the F8F more closely mimics propulsion in daily life or game play there is more variation in real life in length, slope and velocity of wheelchair propulsion, there are different terrains with varying frictional resistance and turns require changing radii. Furthermore, many demanding activities of daily life, like transfers, pressure relief lifts, reaching over head and sport specific activities like throwing and receiving a ball or tackling are missing. Nevertheless, this study focussed on the most repetitive activity of daily life and sports for wheelchair users, namely, wheelchair propulsion. Secondly, it remains unclear at which state of fatigue, influenced by the standardized foregoing parts of the investigation as described earlier and their journey to the movement laboratory, the participants started the F8F. Nevertheless, all participants completed the same assessments prior to the F8F. Thirdly, 8 participants used drugs to treat upper extremity pain in the last 3 months, however none of them had pain that limited their ability to propel. Fourthly important muscles to additionally include would be M. deltoideus pars clavicularis and spinalis. As weakness in the M. deltoideus has shown to be compensated by the rotator cuff muscles and *vice versa* (Slowik et al., 2016) it is important to investigate the influence of fatiguing propulsion on all three anatomical parts of the M. deltoideus. Additionally, shifts in activation ratio between these muscles is seen as a potential cause for decreased joint stability (van Drongelen et al., 2013). Finally, the strict inclusion and exclusion criteria (for example, pain limiting the ability to propel or congenital causes of SCI) may mean the study is not applicable to certain populations.

To gain further knowledge about the influence of fatigue on the development of shoulder pain in people with SCI it is important to develop fatigue protocols that include daily life or sport specific activities, survey their fatiguing effects on the muscular, neural, cardio-vascular and cognitive function and further validate the parameters applied. For validation of existent or future fatigue protocols a gold standard measure for propulsion induced fatigue is needed. Furthermore, the addition of resting HR, peak HR, localised RPE and the assessment of pain before, during and after the protocol is recommended. Also, the development of specific protocols to assess fatigue and its effect during other activities of daily life like pressure relief lifts, overhead activities, reaching and propulsion on uneven ground as well as developing new and adapting existing protocols to the specific demands of individual wheelchair sports is important. Furthermore, the following parameters are recommended to be included in future studies on the topic at hand: temporal onset of muscular fatigue and pain, propulsion technique, wheelchair settings, sitting posture and level of activity in daily life.

The current study is unique in giving clearer insight into the mechanisms of performance fatigability and perceived fatigability throughout the F8F, a protocol that has been used in previous investigations studying the effect of fatiguing wheelchair propulsion (Collinger et al., 2010a; Bossuyt et al., 2020a; Bossuyt et al., 2020c; Bossuyt et al., 2020d; Arnet et al., 2022a). Performance fatigability was shown in a consistent increase throughout the protocol in HR and RMS of the EMG signal of the M. pectoralis major pars sternalis and M. trapezius pars descendens. The subsequent significant consistent increase in RPE demonstrates the effect of the protocol with regards to perceived fatigability.

## Data Availability

The raw data supporting the conclusion of this article will be made available by the authors, without undue reservation. The datasets generated and/or analyzed during the current study are available from the corresponding author on reasonable request.
