# Supplementary figures and images for: Changes in neuromuscular activation, heart rate and rate of perceived exertion over the course of a wheelchair propulsion fatigue protocol
Source: Front Physiol. 2023 Oct 18;14:1220969. doi: 10.3389/fphys.2023.1220969 (PMC10619735; doi:10.3389/fphys.2023.1220969)

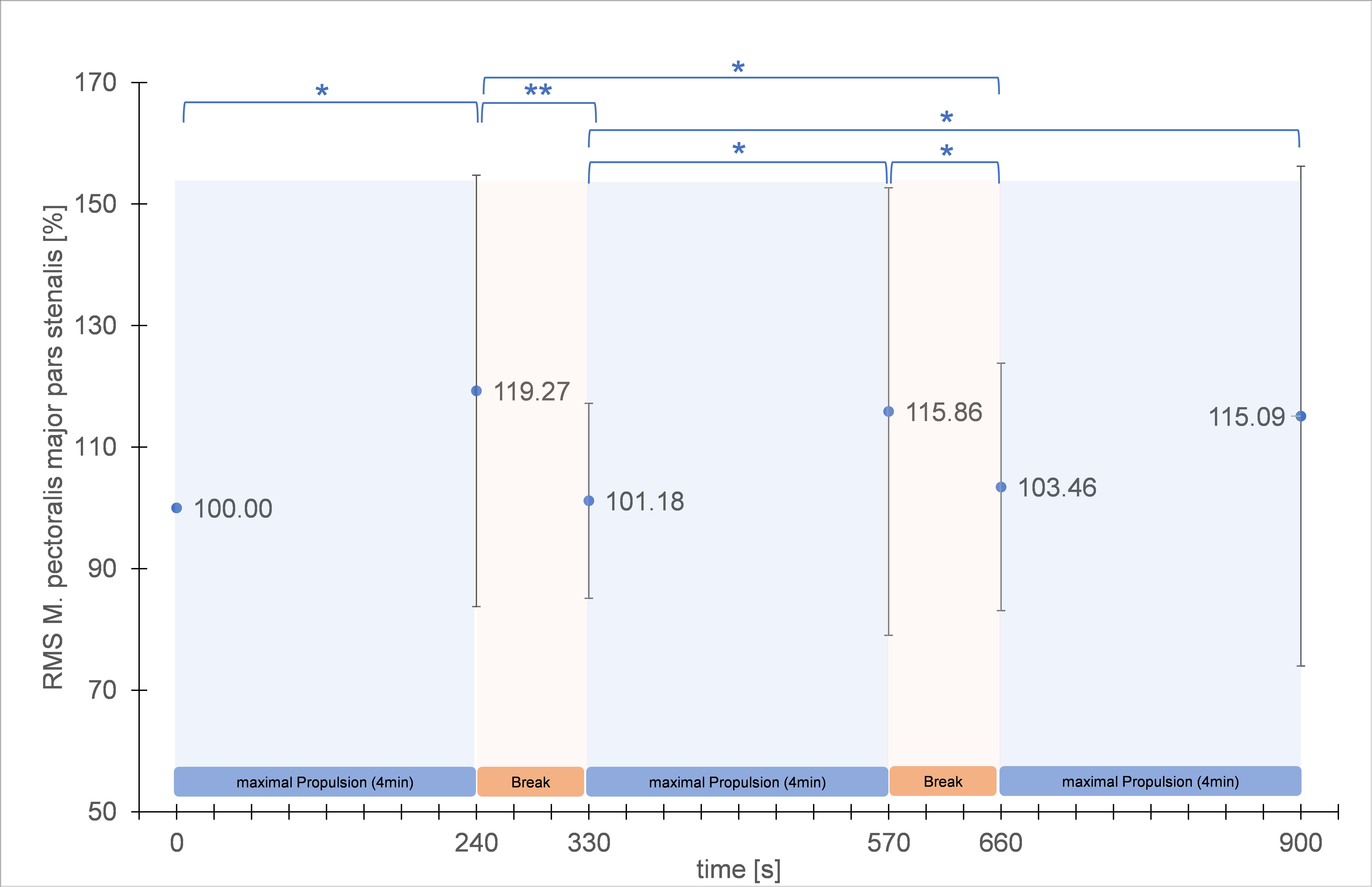

Supplement: Supplementary file 1 [file Image3.JPEG]

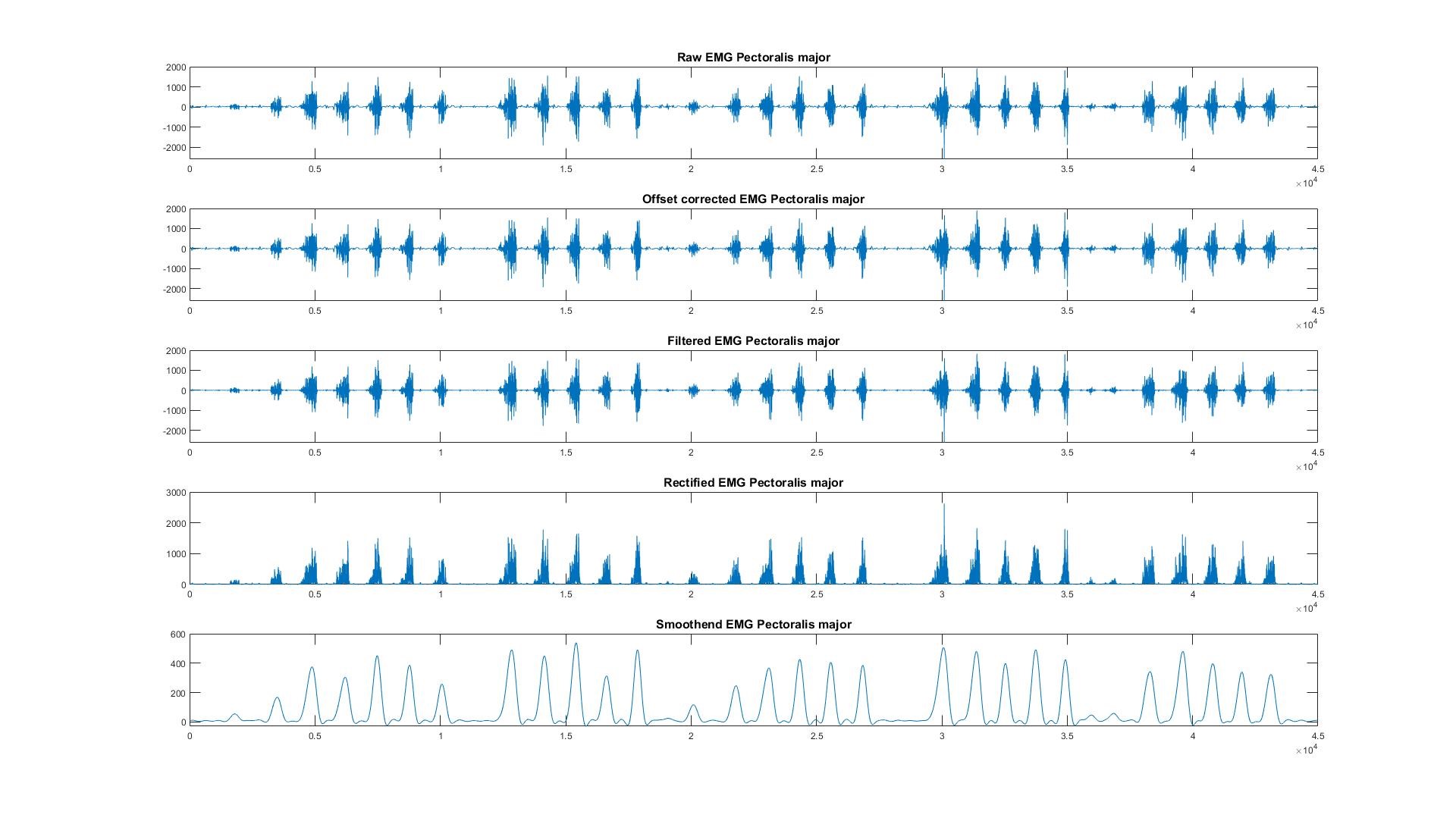

Supplement: Supplementary file 2 [file Image1.JPEG]

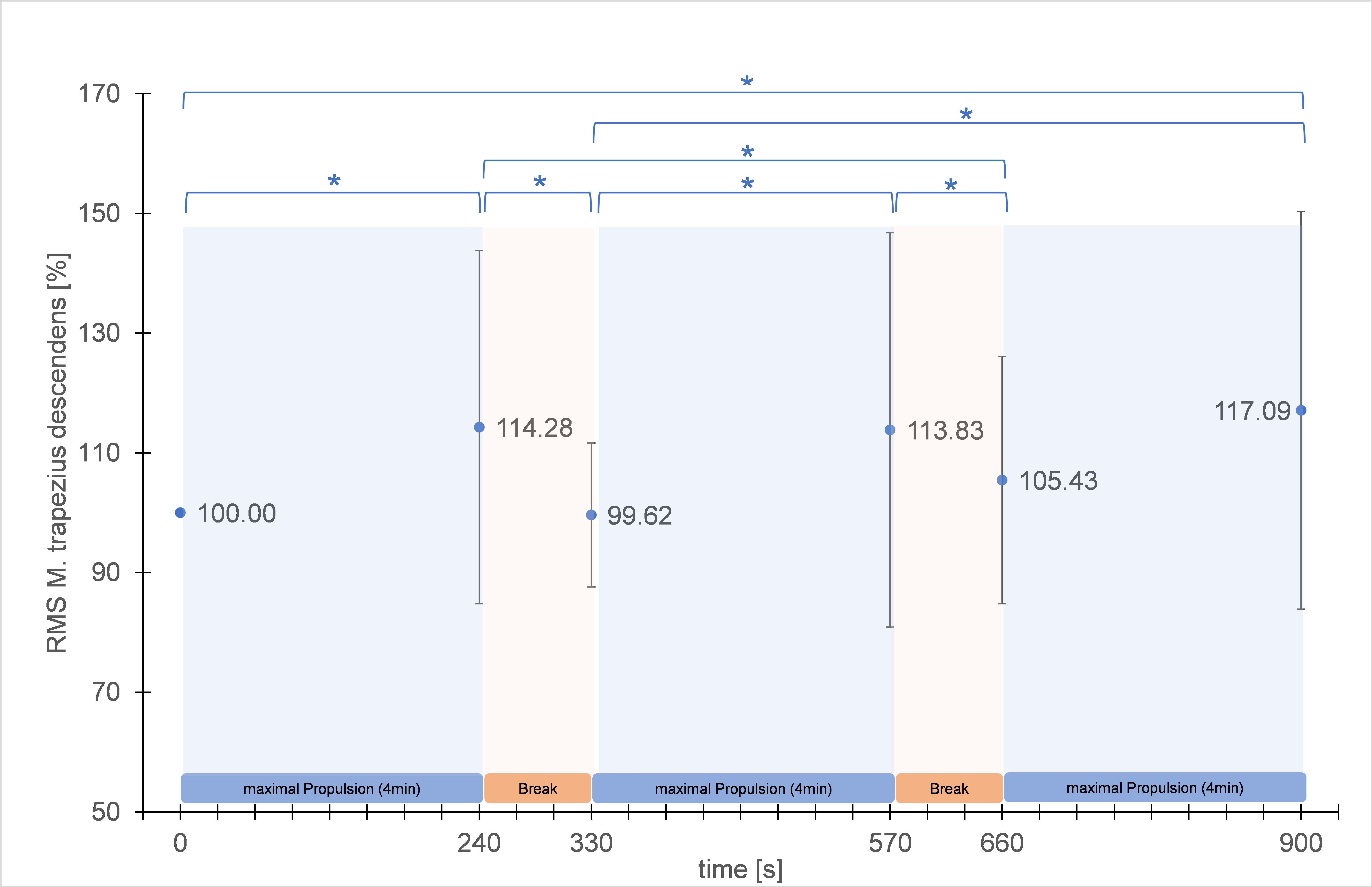

Supplement: Supplementary file 3 [file Image2.JPEG]
